# Passive Fit in Digital Full-Arch Implant Rehabilitation: A Clinical Application of an Adaptive Titanium Base

**DOI:** 10.7759/cureus.117130

**Published:** 2026-09-29

**Authors:** John Heimke, Gregori M Kurtzman, William S Blood

**Affiliations:** 1 Dentistry, Private Practice, Rocky River, USA; 2 General Dentistry and Implantology, Private Practice, Silver Spring, USA; 3 Oral and Maxillofacial Surgery, Private Practice, Westlake, USA

**Keywords:** all-on-4, digital dentistry, full-arch implant rehabilitation, implant prosthodontics, passive fit, zirconia hybrid prosthesis

## Abstract

Passive fit remains a critical factor in the long-term success of implant-supported full-arch prostheses. Although contemporary digital workflows have improved restorative accuracy, minor discrepancies associated with scanning, software alignment, manufacturing tolerances, and prosthetic delivery remain unavoidable and may contribute to biomechanical stress within the implant-prosthetic complex.

This article describes the integration of the NikolasBase/Passivity Plus restorative system within a fully digital All-on-4 mandibular rehabilitation. A 69-year-old male underwent treatment utilizing CBCT (cone beam computed tomography) planning, facial scanning, intraoral digital scanning, ROE ArchBridge verification, CAD/CAM design, and zirconia hybrid fabrication. The NikolasBase/Passivity Plus system incorporates a self-adjusting titanium base with a pivotable cap, conical cold-weld interface, and flexible washer mechanism designed to preserve prosthetic alignment during torque application and compensate for minor restorative discrepancies.

Clinical and radiographic evaluation demonstrated complete seating of the definitive zirconia hybrid prosthesis and successful integration of the adaptive titanium-base components within the digital workflow; passive fit or strain was not directly measured. Adaptive prosthetic connection systems are intended to accommodate small residual restorative discrepancies; however, this single case cannot establish that the system reduces strain, mechanical complications, or biologic complications. While this clinical report demonstrates a predictable restorative workflow, additional long-term clinical studies are required to evaluate the impact of adaptive prosthetic connection systems on mechanical complications and peri-implant tissue stability.

## Introduction

Full-arch implant rehabilitation has undergone substantial evolution with the integration of digital technologies into both the surgical and restorative workflows. Protocols utilizing cone beam computed tomography (CBCT), intraoral scanning, photogrammetry, CAD/CAM manufacturing, facial scanning, and additive manufacturing have improved restorative efficiency, doctor-lab communication, and overall treatment predictability [1-3]. Despite those advancements, achieving passive fit in implant-supported prosthetics remains one of the most significant biomechanical challenges in implant treatment.

Passive fit has traditionally been defined as the absence of strain at the implant-prosthetic interface when the prosthesis is secured to the implants [4]. Inaccuracies during impression capture, digital stitching, manufacturing, framework processing, or prosthetic insertion may create micro-movement and strain within the restorative complex. Even minor discrepancies may transfer stress to prosthetic screws, restorative frameworks, implants, and the surrounding peri-implant bone, which can potentially contribute to screw loosening, component fracture, loss of preload, prosthetic instability, and crestal bone loss over time [4-7]. Long-span full-arch restorations are particularly susceptible due to cumulative distortion across the arch that may magnify those biomechanical effects.

Although digital workflows have significantly reduced restorative inaccuracies compared with traditional analog techniques, complete elimination of distortion remains unrealistic [8,9]. Cross-arch scanning discrepancies, implant divergence, software alignment variability, and manufacturing tolerances continue to contribute to prosthetic misfit. Additionally, tightening forces generated during prosthetic screw fixation may re-center the prosthesis during torque application and negate alignment achieved during seating [6]. As a result, restorative systems capable of compensating for minor discrepancies rather than rigidly resisting them have become increasingly important in contemporary implant prosthetics.

This article reviews the biomechanical significance of passive fit within a contemporary fully digital All-on-X restorative workflow.

## Technical report

NikolasBase/bottom of form

Passivity Plus and Passive Fit in Full-Arch Implant Prosthetics

Passive fit remains a fundamental determinant of long-term success in implant-supported full-arch restorations. Even minimal discrepancies at the implant-prosthetic interface can generate cumulative biomechanical stress that is transferred to the screw joint, prosthetic framework, and surrounding peri-implant bone, increasing the risk of screw loosening, framework fracture, prosthetic complications, and marginal bone loss [10,11]. Although advances in digital workflows, including intraoral scanning, photogrammetry, CAD/CAM fabrication, additive manufacturing, and centralized manufacturing, have significantly improved restorative accuracy, micrometer-level discrepancies remain unavoidable and accumulate throughout the restorative process [12-14]. Consequently, passive fit cannot be assumed to be completely achieved through digital precision alone and instead requires prosthetic systems capable of compensating for these minor inaccuracies rather than resisting them.

The NikolasBase/Passivity Plus was developed to address this long-standing limitation through a self-adjusting titanium base system (Ritter Implants USA, San Antonio, Texas) designed for multi-unit abutment-supported full-arch restorations. According to the manufacturer's technical description, the system incorporates a pivotable, self-adjusting cap designed to permit limited controlled movement during prosthesis seating (reported by the manufacturer as less than 2 degrees) (Figure 1). This is a device-design claim and should not be interpreted as independent clinical evidence that the system reduces prosthetic strain [15]. This allows the prosthesis to align to the true spatial orientation of the implants rather than being forced into position. In contrast, conventional titanium bases tend to re-center during torque application, which may negate the compensatory alignment achieved during seating and introduce stress into the implant-prosthetic complex. The design is intended to preserve the adjusted position obtained during seating; whether this produces a clinically meaningful reduction in strain requires direct biomechanical and prospective clinical evaluation.

**Figure 1:**
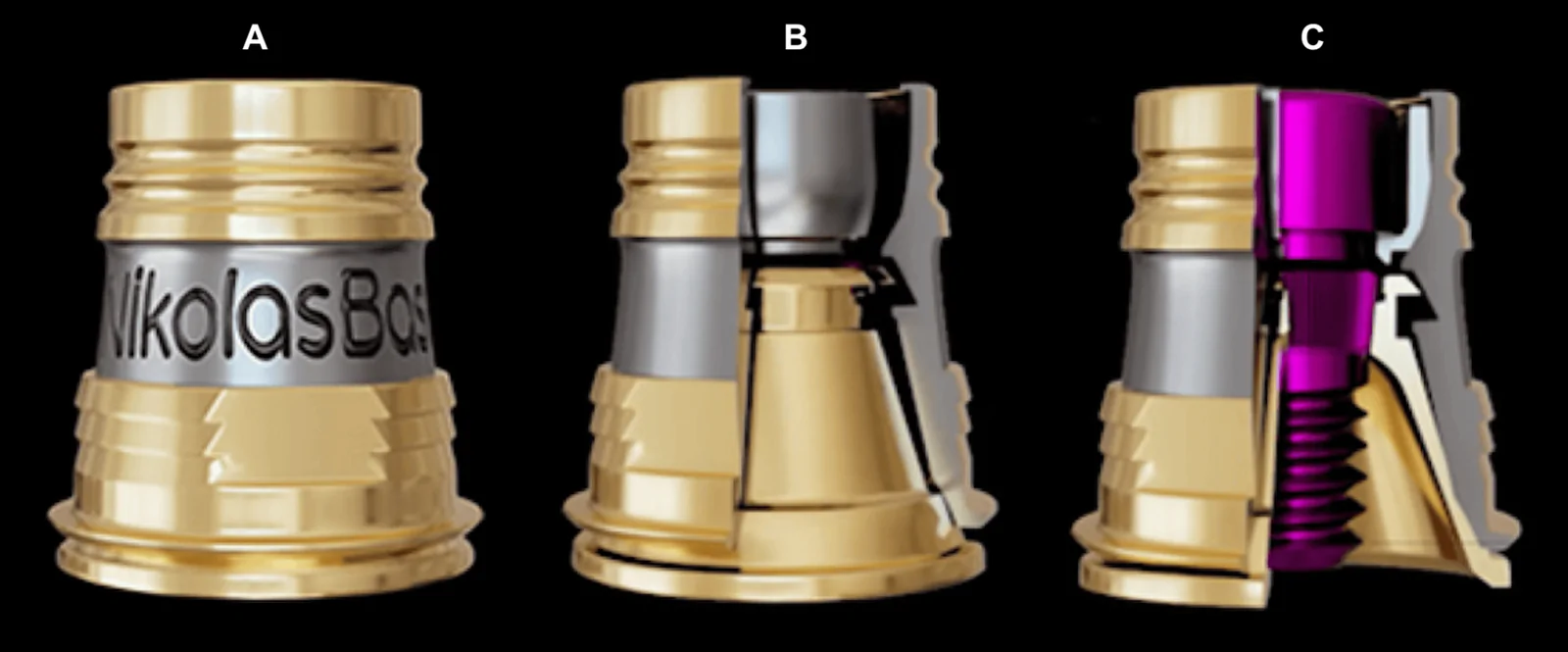
The NikolasBase/Passivity Plus abutment (A), with a cutaway of the self-adjusting cap showing how it engages the underlying abutment (B) and a cutaway of the screw fixating it to the underlying MUA (C).

The manufacturer also describes a conical screw interface with a 5-degree taper that is intended to create frictional engagement during torque application. The proposed effects on preload stability and interface micro-movement are device-specific engineering claims and require citation to the manufacturer's technical documentation; they were not directly measured in this case. Because screw loosening remains one of the most common mechanical complications associated with implant prosthodontics, improved preload maintenance may enhance long-term restorative stability [16,17]. Importantly, the cold-weld interaction remains mechanically reversible, permitting routine prosthetic retrieval and maintenance without permanent deformation of the components.

The system also incorporates a washer mechanism that, according to the manufacturer, is designed to accommodate centering forces generated during screw tightening. In conventional implant systems, tightening forces frequently re-center the prosthesis and eliminate any compensatory alignment achieved during seating, thereby reintroducing stress into the restorative complex. Its proposed ability to preserve an adjusted prosthetic position during torque application is a design rationale and was not independently quantified in the present report. This mechanism functions through three sequential phases: initial micro-adjustment during seating, controlled torque application with force absorption, and final stabilization through the conical cold-weld effect. Those interactions collectively are intended to maintain a mechanically stable yet stress-reduced implant-prosthetic interface.

From a clinical perspective, improved passivity may reduce both biologic and mechanical complications associated with full-arch implant restorations. Reduced interfacial strain has been associated with improved screw joint stability, decreasing the incidence of screw loosening, and reducing crestal bone remodeling around implants [10,11,17]. Additionally, systems capable of compensating for minor restorative discrepancies may decrease the need for prosthesis remakes or extensive chairside adjustments, thereby improving restorative efficiency and reducing treatment complexity [16,18].

Proper clinical protocol remains essential for predictable clinical outcomes. During prosthesis delivery, screws should initially be engaged with minimal turns to allow unrestricted movement of the pivotable caps. Progressive tightening in a cross-pattern sequence permits uniform seating of the prosthesis prior to final torque application. Final torque with the torque wrench should not be applied to any individual screw until all components are engaged with finger use of the wrench tip, allowing the system to achieve its compensatory alignment before stabilization occurs.

It should be emphasized that systems designed to enhance passive fit are intended to compensate for minor restorative discrepancies and are not substitutes for accurate treatment planning, precise data acquisition, appropriate prosthetic design, or verification of fit. When integrated into a controlled restorative workflow, technologies intended to improve passivity may enhance prosthetic stability, reduce mechanical complications, and support long-term success in full-arch implant rehabilitation.

Clinical Workflow and Prosthetic Delivery Protocol

A 69-year-old male patient initially presented for consultation regarding a possible maxillary and mandibular full-arch implant rehabilitation after being informed elsewhere that limited corrective options were available for the existing dentures. The patient reported being a non-smoker. The mandibular arch was selected for treatment at this stage because of financial considerations expressed by the patient. The maxillary arch was not treated during the episode of care reported in this technical report. Clinical and radiographic evaluation revealed severe maxillary atrophy with less severe mandibular resorption (Figure 2A). A CBCT evaluation was prescribed to facilitate digital treatment planning for an All-on-X fixed hybrid reconstruction of the mandibular arch (Figure 2B). Initial records included facial scanning, intraoral digital scanning with the Medit IOS system (Medit USA, Inc., Newport Beach, California), RayFace (RayFace America, Inc., Fort Lee, New Jersey) facial acquisition, 2D digital smile design analysis, photographs, and video documentation. Diagnostic planning established a proposed zirconia full-arch hybrid prosthesis with a target shade in the BL1-BL3 range.

**Figure 2:**
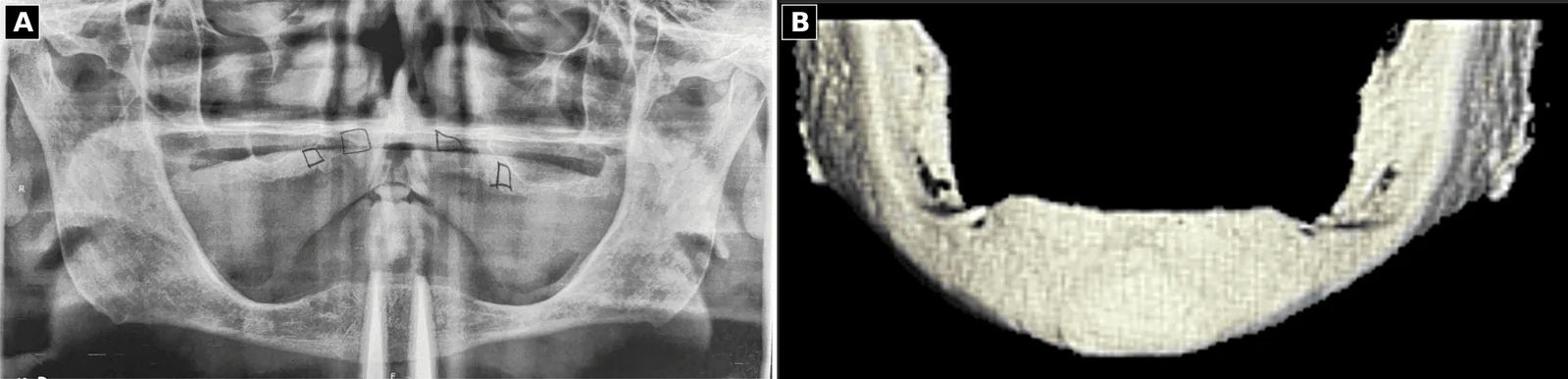
Pretreatment radiographic assessment. (A) Panoramic radiograph demonstrating the edentulous arches, severe maxillary ridge atrophy, and less severe mandibular resorption. (B) Frontal CBCT reconstruction of the mandible demonstrating residual ridge anatomy and available bone volume used for digital planning of the four-implant fixed full-arch reconstruction.

Surgical treatment was coordinated with the oral surgeon utilizing a fully digital workflow. Four Straumann BLX RP implants (3.75 mm × 12 mm) (Straumann USA, LLC, Andover, Massachusetts) were placed in the mandibular arch under general anesthesia. Multiunit abutments with a 2.5 mm collar height were delivered at the time of surgery; plastic healing caps were then placed, and the flaps were secured with sutures (Figure 3A). Grammetry verification utilizing the Roe ArchBridge protocol (ROE Dental Lab, Independence, Ohio) was completed in conjunction with Medit IOS scanning to improve cross-arch accuracy during full-arch digital acquisition.

**Figure 3:**
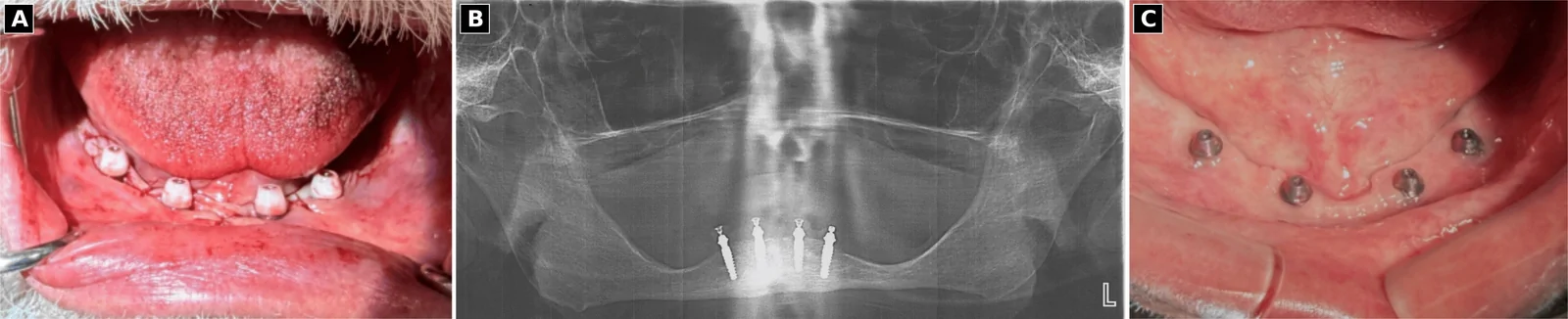
Implant placement and early healing. (A) Clinical view after placement of the four mandibular implants and multiunit abutments, with plastic healing caps in place and the flaps secured with sutures. (B) Panoramic radiograph at the one-month postoperative evaluation demonstrating implant position and early peri-implant healing. (C) Clinical view of the mandibular implants and healed soft tissues after the removal of the plastic healing copings.

The ROE ArchBridge protocol is a laboratory-assisted digital verification workflow developed by ROE Dental Laboratory to improve full-arch implant scanning accuracy in All-on-X restorations where cross-arch distortion may compromise prosthetic passivity. The system utilizes unique scan bodies, with no two alike, which prevents scanner ghosting and digital transition from one to the other during intraoral scanning, reducing cumulative stitching errors associated with long-span edentulous arches. By stabilizing implant positional relationships during digital acquisition, the workflow improves transfer accuracy in cases with divergent implants, extended arch spans, or limited anatomic landmarks. The protocol typically includes placement of multiunit abutments followed by ArchBridge scan bodies, intraoral scanning, and laboratory merging of data sets to generate the definitive digital master model. Often utilized with high-accuracy intraoral scanners such as the Medit i900. The ROE ArchBridge protocol allows for records similar to traditional splinted open-tray analog impressions by enhancing cross-arch accuracy, improving passive fit, and potentially reducing screw loosening, prosthetic strain, and chairside adjustment requirements in definitive zirconia full-arch restorations. A printed interim healing hybrid prosthesis is fabricated and delivered immediately following surgery to maintain function, soft tissue support, and esthetic continuity during osseointegration.

Postoperative evaluation at one month and a radiograph were taken, as shown in Figure 3B. This demonstrated satisfactory early healing and favorable patient adaptation to the interim prosthesis. Healing caps were removed during the early postoperative phase (Figure 3C), and the printed hybrid prosthesis was secured utilizing DESS prosthetic screws (DESS USA, Lake Mary, Florida). Occlusion was refined, and access openings were sealed with PTFE tape. The patient reported a favorable esthetic response to the provisional restoration. Oral hygiene and postoperative dietary instructions were reviewed, including implementation of a maintenance protocol and soft tissue management regimen during healing.

The interim healing restoration was removed. A Medit intraoral scan was obtained both with and without healing caps to verify implant position and soft tissue architecture. Prototype verification was subsequently performed utilizing a printed All-on-4 hybrid try-in prosthesis. Best results were obtained with the prototype with NikolasBase/Passivity Plus copings added to the prototype to have a "True-Prototype" to verify fit. The printed prototype hybrid prosthesis was seated and adjusted, with screw tightening completed prior to esthetic evaluation. The prototype was evaluated for vertical dimension, occlusion, esthetics, phonetics, and overall prosthetic design. Occlusal discrepancies were corrected chairside, and the access openings were sealed with PTFE tape pending definitive fabrication. The patient was appointed to allow them to test drive the prosthesis to approve the function and esthetics or indicate changes needed. The patient returned to review their thoughts on the prototype, indicating they were happy with the esthetics and had no functional complaints. ROE Dental Laboratory was informed that no changes were needed and to proceed with fabrication of the final zirconia hybrid prosthesis.

Following verification of the prototype restoration, the definitive laboratory workflow was completed utilizing a fully digital CAD/CAM protocol integrated with the NikolasBase/Passivity Plus restorative system. The mandibular arch scan data was imported into Exocad software (Exocad America, Inc., Wakefield, Massachusetts) to establish the definitive digital working model and prosthetic design parameters (Figure 4). The virtual design of the mandibular hybrid prosthesis was then completed with evaluation of both the prosthetic contours and the underlying soft tissue architecture to optimize emergence profile, cleansability, and restorative support (Figure 5A). Articulated virtual scans were subsequently reviewed to verify occlusal relationships, prosthetic space, and overall restorative positioning prior to final fabrication (Figure 5B).

**Figure 4:**
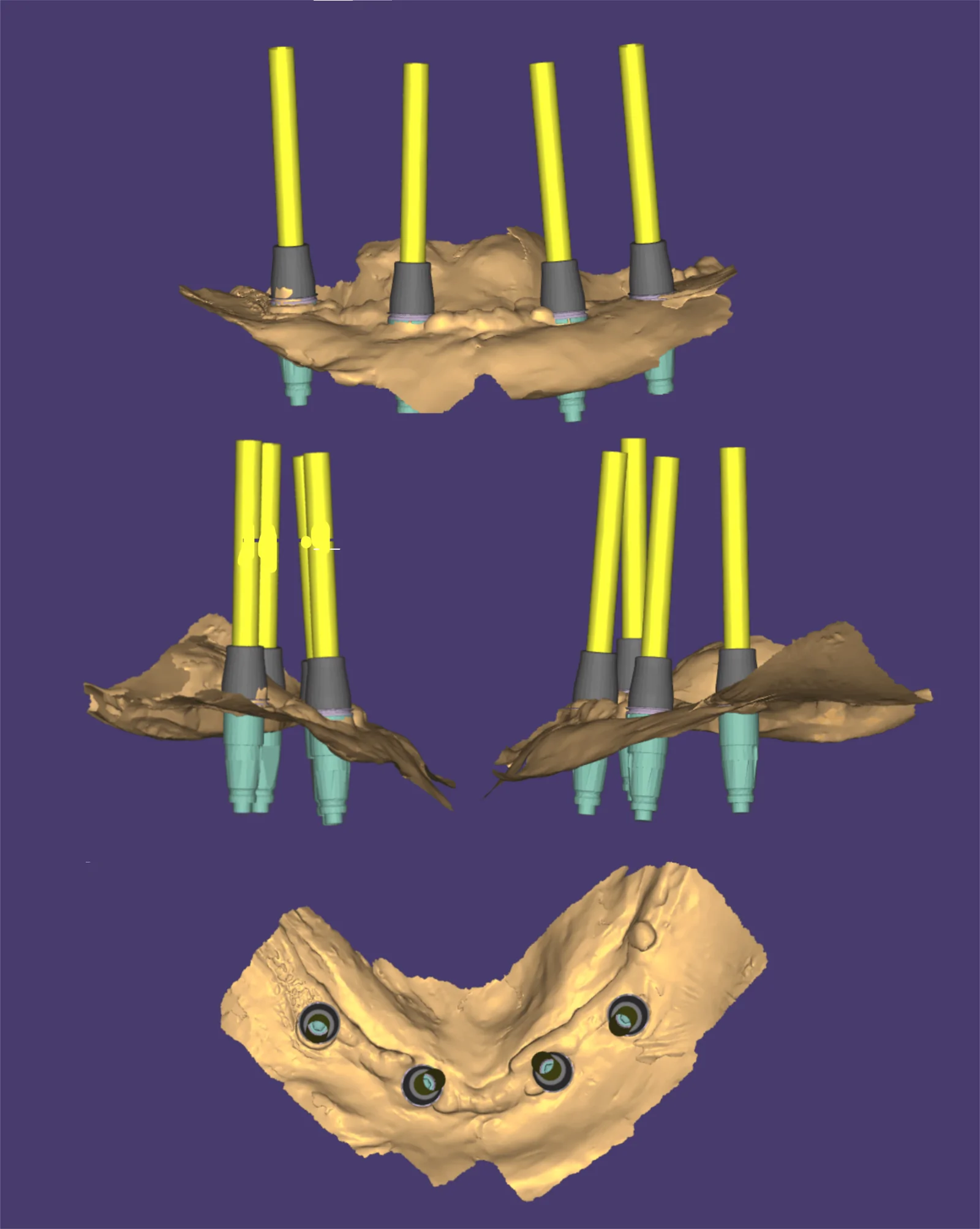
Virtual mandibular arch following intraoral scanning and importation into Exocad software (Exocad America, Inc., Wakefield, Massachusetts) for definitive digital prosthetic planning.

**Figure 5:**
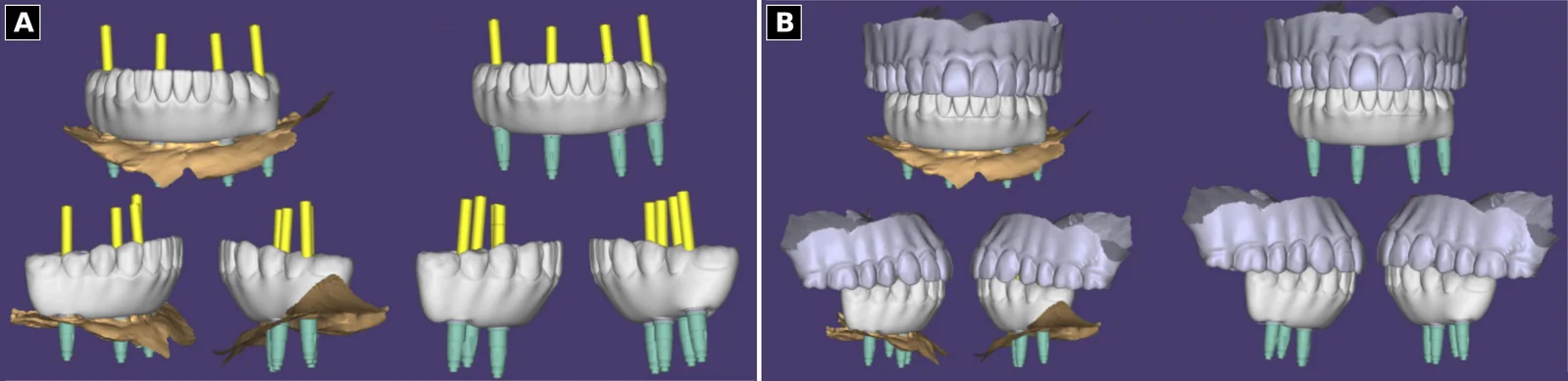
Digital design of the mandibular full-arch hybrid. (A) Virtual design with the soft-tissue scan displayed and removed to evaluate prosthetic contours and tissue relationships. (B) Articulated virtual scans used to assess occlusion, restorative space, and overall prosthetic positioning.

Digital prosthetic design also permitted precise positioning of the NikolasBase/Passivity Plus abutments within the zirconia hybrid while maintaining appropriate screw access channel orientation and restorative material thickness (Figure 6). The relationship of the NikolasBase/Passivity Plus components to the multiunit abutments was evaluated virtually prior to milling to confirm prosthetic alignment and restorative passivity within the digital environment (Figure 7).

**Figure 6:**
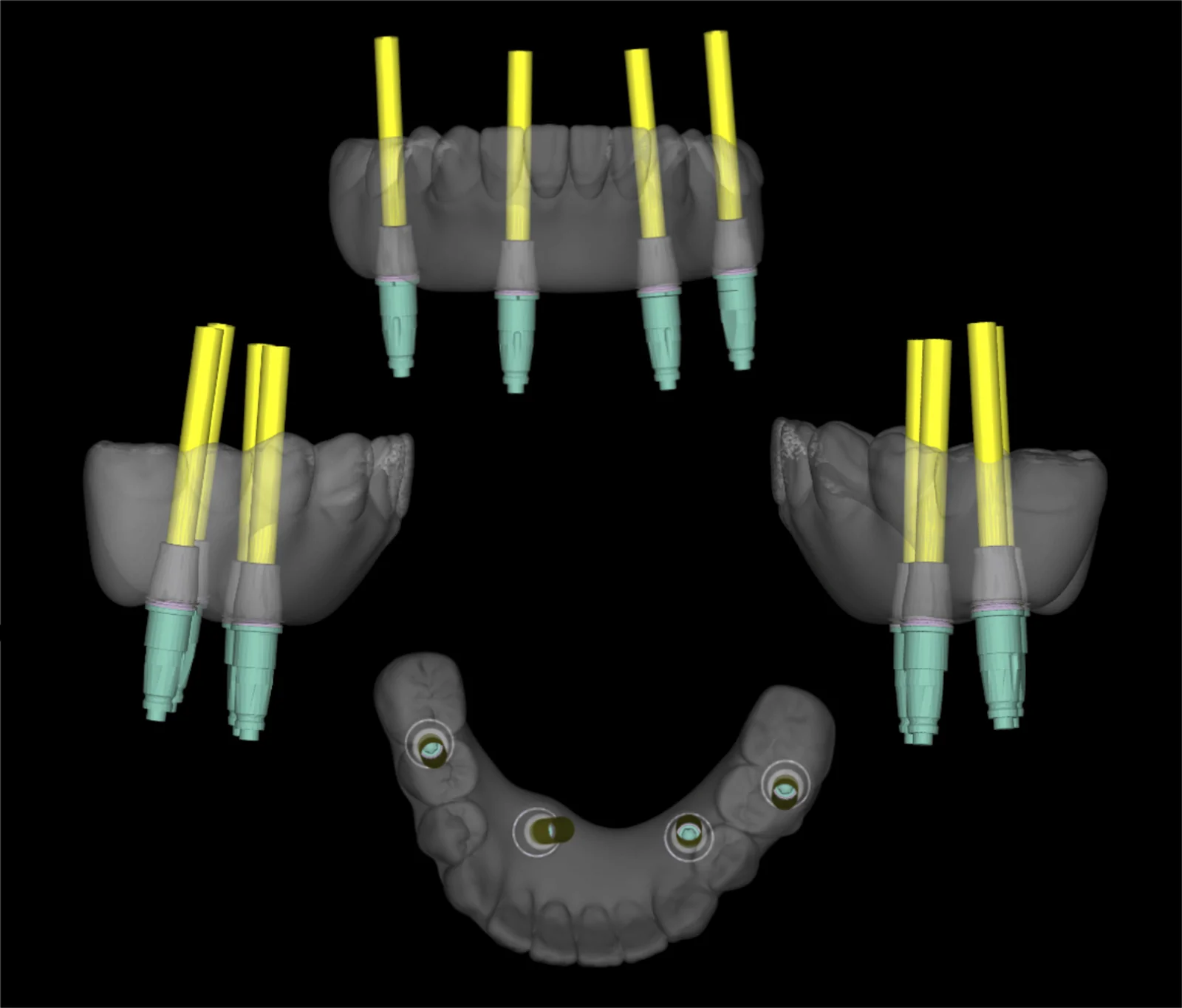
Virtual mandibular hybrid demonstrating the planned positioning of the NikolasBase/Passivity Plus abutments and screw-access channels within the prosthesis.

**Figure 7:**
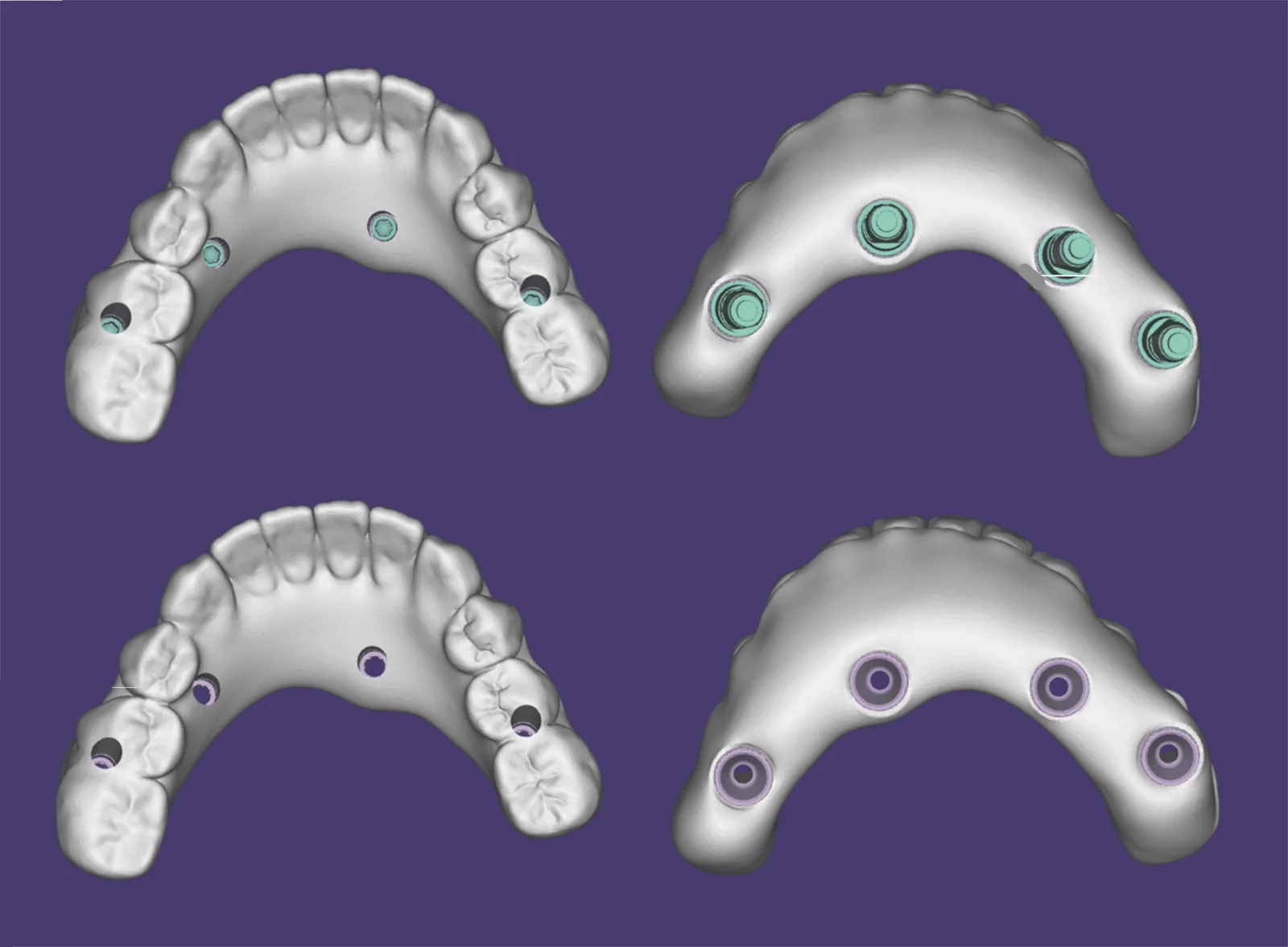
Virtual views of the designed mandibular hybrid showing the relationship of the NikolasBase/Passivity Plus abutments to the multiunit abutments and their position within the prosthesis before definitive fabrication.

The definitive mandibular hybrid prosthesis was then milled from zirconia utilizing CAD/CAM manufacturing technology (Figure 8). Following milling and finishing, the NikolasBase/Passivity Plus abutments were prepared for luting into the definitive zirconia framework (Figure 9A). The abutments were subsequently secured within the prosthesis according to the laboratory protocol to create the definitive screw-retained hybrid restoration (Figure 9B). Final finishing and polishing were then completed prior to clinical delivery of the definitive mandibular zirconia hybrid prosthesis (Figure 9C).

**Figure 8:**
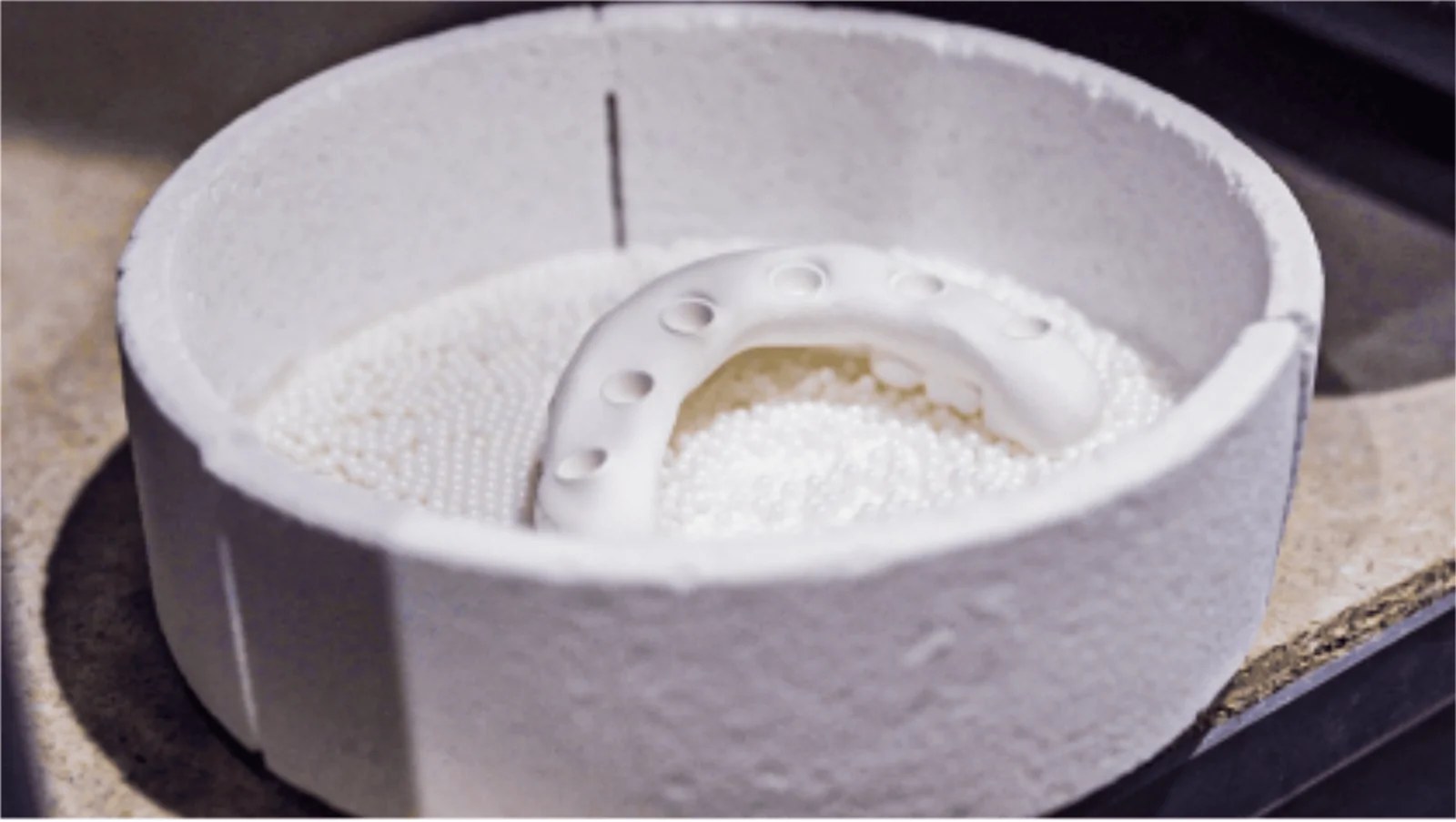
Milled mandibular zirconia hybrid before incorporation of the adaptive titanium-base components and final finishing.

**Figure 9:**
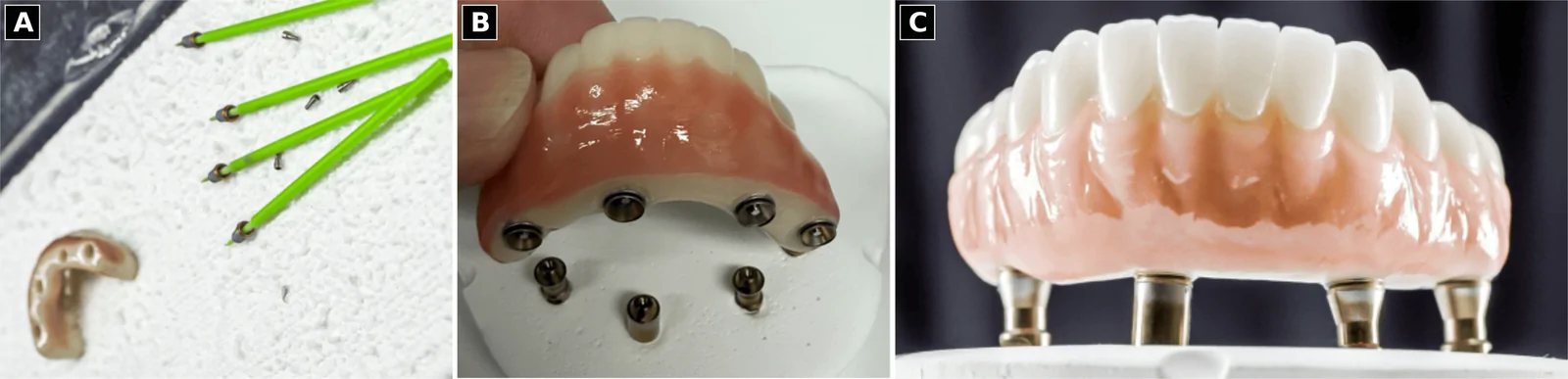
Laboratory integration and completion of the definitive prosthesis. (A) Preparation for luting the NikolasBase/Passivity Plus abutments into the zirconia hybrid. (B) Hybrid prosthesis after incorporation of the NikolasBase/Passivity Plus abutments. (C) Finished definitive mandibular zirconia hybrid ready for clinical insertion.

The digital workflow utilized in this case permitted integration of surgical planning, restorative design, prosthetic verification, and CAD/CAM manufacturing within a coordinated restorative sequence. This was intended to improve prosthetic accuracy, restorative efficiency, and passive fit of the definitive full-arch implant prosthesis.

The patient presented, and the definitive lower zirconia All-on-4 hybrid bridge was delivered. Prosthetic fixation screws were torqued to 15 Ncm according to the restorative protocol. Radiographs were taken to confirm complete clinical and radiographic seating of the NikolasBase/Passivity Plus abutments in the zirconia hybrid to the implants intraorally (Figure 10). Passive fit and prosthetic strain were not quantitatively measured. Occlusion was refined to establish bilateral stability with minimized cantilever stress during excursive function. Screw access channels were filled with PTFE tape and sealed utilizing LuxaFlow composite resin (DMG America, LLC, Ridgefield Park, New Jersey). The final prosthesis followed the contour of the mandibular ridge (Figure 11A) and matched the occlusion of the opposing arch (Figure 11B). Final photographs and video documentation were obtained, and both written and verbal postoperative instructions were reviewed with the patient. A structured maintenance interval consisting of prophylaxis visits at three-month intervals was prescribed to support long-term peri-implant soft tissue stability and prosthetic maintenance.

**Figure 10:**
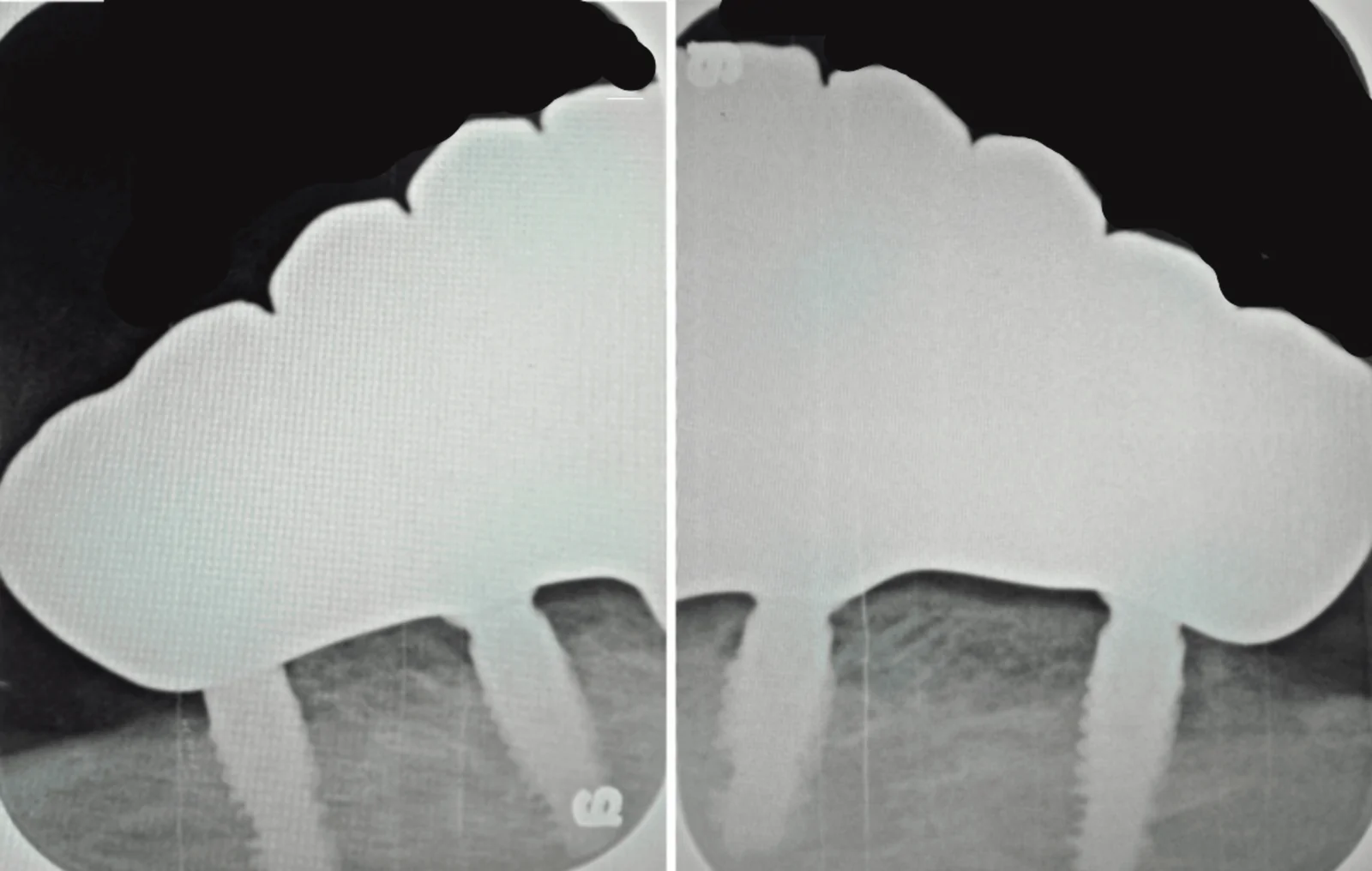
Radiographs taken to confirm seating of the NikolasBase/Passivity Plus abutments in the zirconia hybrid to the implants.

**Figure 11:**
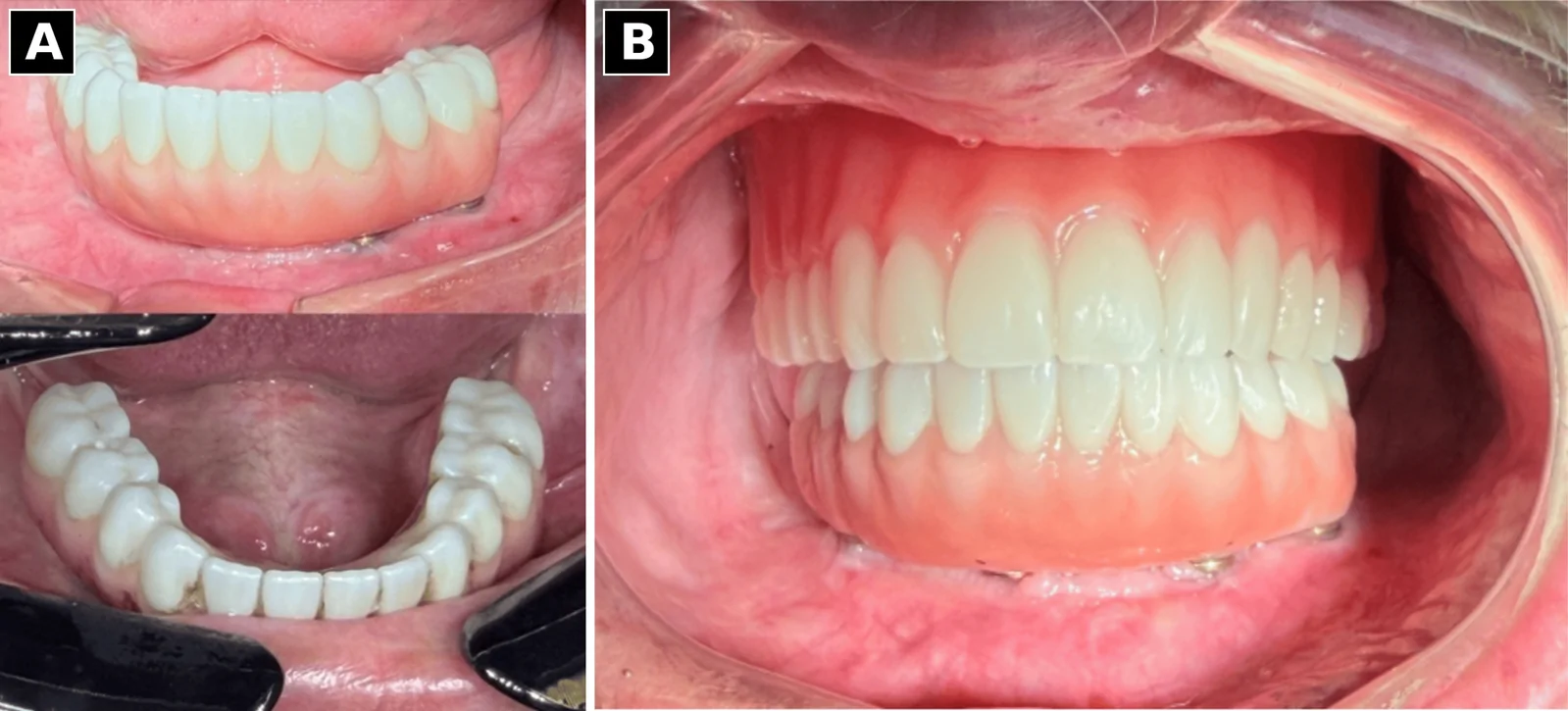
Clinical delivery of the definitive restoration. (A) Inserted NikolasBase/Passivity Plus mandibular zirconia hybrid demonstrating adaptation to the mandibular ridge. (B) Frontal clinical view of the definitive mandibular hybrid in occlusion with the opposing arch.

The digital workflow utilized in this case permitted integration of surgical planning, restorative design, prosthetic verification, and CAD/CAM manufacturing within a coordinated restorative sequence. This was intended to improve prosthetic accuracy, restorative efficiency, and passive fit of the definitive full-arch implant prosthesis.

## Discussion

Passive fit remains an important biomechanical objective in full-arch implant prosthetics because discrepancies at the implant-prosthetic interface can introduce strain into the framework and screw joint. Earlier reviews emphasized that an absolutely passive framework is difficult to obtain with available clinical and laboratory procedures, while later systematic reviews found that the clinical consequences of misfit are complex and that a universally accepted threshold for clinically tolerable misfit has not been established [5,11,17]. These findings support a practical objective of minimizing misfit rather than assuming that any digital or analog workflow can eliminate it completely.

The present case is consistent with the literature showing that digital implant impressions can provide clinically useful accuracy in edentulous arches, but that complete-arch acquisition remains technique-sensitive. Papaspyridakos et al. reported digital implant impressions to be comparable in accuracy with splinted conventional impressions in an edentulous model [9]. A subsequent systematic review and meta-analysis likewise found digital implant impressions to be a viable alternative to conventional techniques [19]. More recent evidence focused specifically on full-arch implant prostheses found clinically acceptable intraoral-scan accuracy, while also demonstrating that implant configuration, scanner-related factors, and study methodology influence the measured deviation [20]. These data support the use of digital acquisition in the present workflow while also explaining why an additional cross-arch verification step was incorporated rather than relying on intraoral scanning alone.

The ROE ArchBridge verification step used in this case also parallels the broader literature on auxiliary methods intended to improve complete-arch digital scan accuracy. A systematic review of techniques for complete-arch implant scanning concluded that scan-body position can be distorted during intraoral acquisition and evaluated multiple strategies intended to improve accuracy [21]. More recent systematic review evidence has similarly identified scan-body design and position, inter-implant distance, scan span, scanning technique, scanner type, and the use of scan aids as variables that may affect virtual implant-position transfer [22]. The ArchBridge workflow in this case should therefore be viewed as a verification aid within that broader effort to control cumulative cross-arch error; this case report does not establish that it is superior to other verification or photogrammetry approaches.

The adaptive titanium-base concept used here addresses a different stage of the accuracy chain. Digital scanning and cross-arch verification are intended to improve the transfer of implant position, whereas the NikolasBase/Passivity Plus components are intended to accommodate small residual discrepancies during prosthesis seating and torque application. This distinction is clinically relevant because CAD/CAM fabrication can improve reproducibility but does not guarantee strain-free seating. Karl and Taylor demonstrated that CAD/CAM material and fabrication choices influence strain development in implant-supported frameworks [6], and Abduo and Judge concluded that framework misfit has measurable biomechanical sequelae even though the direct relationship with long-term biologic complications remains incompletely defined [11]. In the present case, complete clinical and radiographic seating was obtained without extensive chairside correction; however, a single case cannot determine whether the adaptive connection itself reduced strain or complication risk.

The findings should also be interpreted in the context of longer-term clinical evidence. Katsoulis et al. found insufficient evidence to define a specific degree of misfit that predicts biologic failure, although screw-related technical complications may be somewhat more frequent when misfit is present [17]. This is important because it prevents overinterpreting the favorable result in this patient. The absence of seating problems at delivery demonstrates the feasibility of the combined workflow, but it does not establish the superiority of the NikolasBase/Passivity Plus system or prove that it will reduce screw loosening, zirconia fracture, marginal bone changes, or other complications over time.

Overall, this case connects with prior research in two complementary ways: contemporary digital acquisition can achieve clinically acceptable full-arch accuracy, while verification and prosthetic strategies remain important because small errors can accumulate across a long span [9,19-22]. The layered workflow used here - digital planning and scanning, cross-arch verification, prototype evaluation, CAD/CAM zirconia fabrication, and an adaptive titanium-base connection - was intended to control error at successive stages rather than depend on a single technology. Prospective comparative studies with quantitative fit or strain measurements and longer follow-up are needed to determine whether this combined approach produces measurable advantages over established digital full-arch protocols.

This technical report has several limitations. It describes a single patient and does not include a control or comparison group. Passive fit was not quantified with strain gauges, photogrammetric deviation analysis, or another objective biomechanical method; clinical and radiographic seating therefore cannot be interpreted as proof of a strain-free interface. In addition, the report does not provide prospective long-term follow-up sufficient to determine whether the adaptive titanium-base system alters the incidence of screw loosening, prosthetic fracture, marginal bone change, or other mechanical or biologic complications. Device-specific mechanical features are based on the manufacturer's design description and should be distinguished from independently demonstrated clinical outcomes. Prospective comparative studies with quantitative fit/strain measurements and longer follow-up are needed.

The patient provided written informed consent for the treatment described in this report. Because this report describes routine clinical care involving a single patient and no experimental intervention, institutional review board review was not required.

## Conclusions

Passive fit remains a critical biomechanical objective in full-arch implant prosthetics despite ongoing advancements in digital restorative workflows. Minor discrepancies introduced during scanning, manufacturing, and prosthetic delivery may generate cumulative stress within the implant-prosthetic complex capable of contributing to biologic and mechanical complications over time. The NikolasBase/Passivity Plus system represents an adaptive titanium-base concept intended to compensate for these discrepancies through controlled prosthetic micro-adjustment, preload stabilization, and preservation of prosthetic alignment during torque application.

Within the fully digital workflow presented, the incorporation of the NikolasBase/Passivity Plus system with cross-arch verification and CAD/CAM zirconia fabrication permitted complete clinical and radiographic seating of the definitive mandibular prosthesis. This technical report demonstrates the feasibility of integrating an adaptive titanium-base system into a digital full-arch workflow, but it does not establish that the system improves passive fit, reduces strain, or decreases mechanical or biologic complications. Additional long-term clinical investigation remains necessary to further evaluate the biomechanical and restorative implications of adaptive prosthetic connection systems in full-arch implant dentistry.
